# Farmers’ knowledge, attitudes, and practices toward animal welfare across different beef cattle farming systems in Phayao Province, Thailand

**DOI:** 10.5455/javar.2025.l948

**Published:** 2025-09-08

**Authors:** Nattamaporn Kongmuang, Payungsuk Intawicha, Surinthip Sakphoowadon, Sureeporn Saengwong

**Affiliations:** 1 School of Agriculture and Natural Resources, University of Phayao, Phayao, Thailand; 2 School of Information and Communication Technology, University of Phayao, Phayao, Thailand

**Keywords:** Animal welfare, extensive, intensive, KAP, semi-intensive

## Abstract

**Objective:**

This study aimed to evaluate the knowledge, attitudes, and practices (KAP) toward animal welfare principles of beef cattle farmers using different farming systems in Phayao Province, Thailand.

**Materials and Methods:**

A structured questionnaire survey was conducted among farmers using extensive (*n* = 20), semi-intensive (*n* = 20), and intensive systems (*n* = 20). Descriptive statistics were employed to describe the demographic characteristics and KAP. Chi-square tests were performed to evaluate the relationships between farming systems and KAP variables. The multivariate influences of the farming system on the overall KAP scores were assessed using a multivariate analysis of variance (MANOVA). Pearson’s correlation coefficients were used to examine the relationships among the KAP components.

**Results:**

The *chi*-square test revealed significant differences (*p* < 0.001) in KAP among farming systems. Intensive farmers showed the highest levels of knowledge (100% good), positive attitudes (55%), and very good practices (80%). However, extensive farmers exhibited lower knowledge (45%), moderate attitudes (90%), and poor practices (95%). MANOVA found that KAP levels were significantly influenced by farming systems (*p* < 0.001). Pearson’s correlation analysis showed significant positive correlations among the KAP components. Attitudes strongly influenced welfare practices (*r* = 0.93, *p* < 0.001), while knowledge revealed positive correlations with both attitudes (*r* = 0.73, *p* < 0.001) and welfare practices (*r* = 0.69, *p* < 0.001).

**Conclusion:**

This study highlights the importance of specific programs, including farmer education, training, and infrastructure development, for improving animal welfare compliance across several farming systems.

## Introduction

Animal welfare is a fundamental component of sustainable livestock production that directly influences the health, productivity, and financial sustainability of cattle. Appropriate welfare practices promote ethical treatment, reduce disease risk, and improve the overall quality of beef products, which increases farm efficiency and profitability. Increasing consumer awareness and regulations globally have made welfare-friendly farming practices essential; consequently, livestock farmers consider animal welfare a priority in their management strategies [1]. Production systems of beef cattle in Thailand are defined as extensive, semi-intensive, and intensive, which raise various issues in the implementation of welfare standards, particularly in smallholder farmers; their knowledge, attitudes, and practices (KAP) concerning animal welfare remain inadequate. However, due to the raised awareness in animal welfare, farming practices, productivity, and their economic sustainability are also changing greatly under the pressure of a rising consumer demand for livestock products that are produced ethically [2]. Through this transition, the moral implications of sustainable farming processes that boost efficiency and competitiveness in the market in the long run are also emphasized [3–5]. Additionally, the application of the animal welfare concept in the farm sector remains a significant challenge, especially among smallholder farmers who lack knowledge, attitudes, and resources [6].

Several factors demonstrate weaknesses in welfare management. Another reason is ignorance of animal welfare principles, which leads to unsuitable management concerning the handling of beef cattle. Limited access to resources, including access to grazing facilities, inadequate watering and feeding, and a lack of funds to invest in physical structures that facilitate welfare, also make it difficult for farmers to adopt welfare-friendly practices as much as they should. The attitude of the farmers is also an essential element, as they prioritize the maximum rate of production over animal welfare considerations [7].

The KAP framework provides a valuable tool for determining the opinions and behaviors of farmers in relation to animal welfare, enabling detailed familiarity with these issues. Although the KAP framework was created to study animal welfare and management, today the research field tends to expand its use to study other aspects, such as public health and educational research [8]. The knowledge part evaluates farmers’ knowledge of the principles of welfare and their related opinions and perceptions, whereas the practice part evaluates welfare-friendly management practices. The KAP structure is a favorable method for determining awareness gaps, obstacles, and opportunities for best practices and the strategies of interventions in livestock systems.

A review of the literature demonstrates that several factors affect farmers’ perspectives on animal welfare, which are quite complex [9]. Similarly, industrial sustainability is frequently affected by consumer concerns and opinions about animal welfare [1]. Therefore, implementing appropriate strategies to improve animal welfare in the beef cattle sector depends on a thorough awareness of the KAP of key stakeholders.

This study employed the KAP framework to examine and compare the KAPs of beef cattle farmers in Phayao Province across different farming systems (extensive, semi-intensive, and intensive). Policies expected to improve animal welfare are applied differently by farmers depending on their management approach, available resources, and market demand [10]. Moreover, there is an inadequate empirical investigation concerning KAP for animal well-being across several farming systems in Phayao Province. Understanding these variations will develop focused procedures, enhance policies, and support training programs that encourage animal welfare compliance, depending on an awareness of these differences. This study aimed to evaluate the KAP of beef cattle farmers in Phayao Province based on animal welfare principles. The objectives of this study were to (1) assess the level of farmers’ KAPs of animal welfare principles across extensive, semi-intensive, and intensive farming systems and (2) identify the factors affecting the adoption of welfare measures and propose strategies for improvement.

The findings will assist in understanding the obstacles and the likelihood of increasing animal welfare in beef cattle production. Policymakers, extension officers, and livestock stakeholders will find significant benefits in the information acquired to create welfare awareness programs and supporting systems for sustainable livestock farming.

## Materials and Methods

### Ethical approval

The Human Ethics Committee and Animal Ethics Committee of the University of Phayao approved this study protocol. The Human Ethics Committee approved this study (number HREC-UP-HSST 1.2/012/68). Following this approval, we conducted a household survey from July to November 2024 involving in-person interviews with 60 beef cattle farmers in Phayao Province. Before participation, all respondents were fully informed of the study’s objectives, data collection procedures, and confidentiality measures. Written informed consent was obtained from each participant to ensure that their involvement was voluntary and that they had the right to withdraw at any stage without any consequences.

### Study area

**Figure 1.**
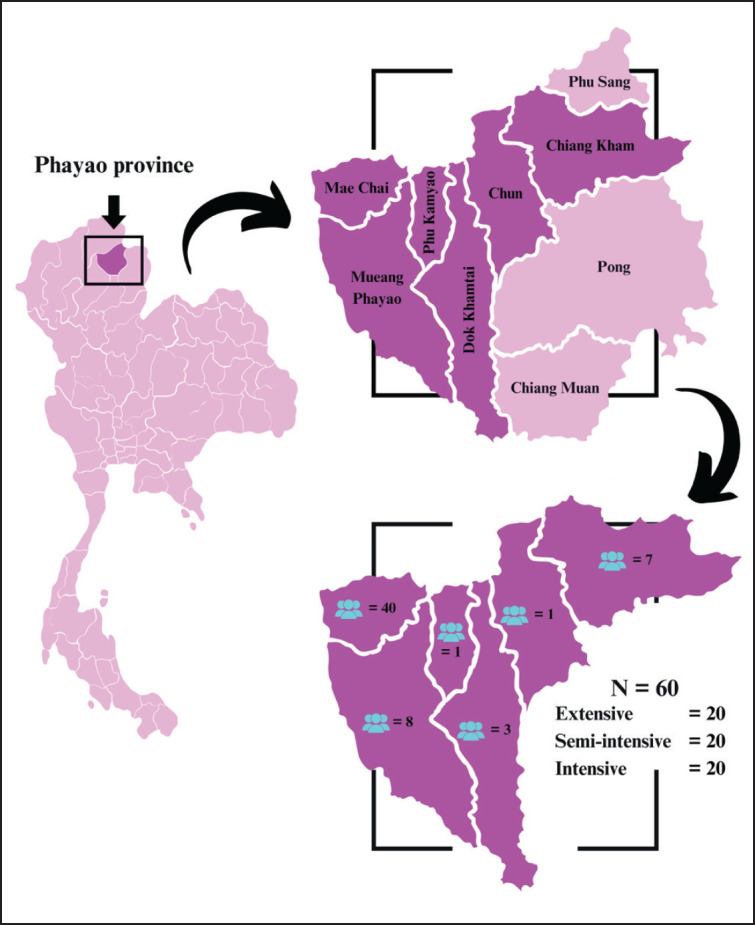
Location of the study area in Northern Thailand.

This study was conducted in northern Thailand’s Phayao Province, which is known for its various cattle-rearing systems. Phayao is an important agricultural region where raising beef cattle is essential for the way people live in rural communities (Fig. 1). The province provides a suitable context for assessing evolving animal welfare KAPs due to the extensive, semi-intensive, and intensive farming systems. The study consisted of several Phayao districts that implement different farming practices.

(1) *Extensive system:* It is a low-input, traditional grazing system in which cattle graze on natural pastures under limited additional feeding and management. Housing is minimal, with cattle frequently remaining in open fields or shelters.

(2) *Semi-intensive system:* It is a hybrid system in which cattle graze while receiving supplemental feed, growth rate enhancement, and herd management. The housing comprises enclosed paddocks or basic barns for shelter against inclement weather and predators.

(3) *Intensive system:* It is a high-input commercial feedlot system characterized by monitored feeding, strict health management, and rapid growth. Cattle are accommodated in carefully constructed stalls or feedlots equipped with adequate ventilation, flooring, and waste treatment equipment.

### Survey procedure and participants

This survey was designed for an equal distribution of farmers among the three types of beef cattle systems in Phayao Province. The study population included beef cattle producers operating under several management strategies. We used a purposive sampling method to select 60 farmers, with 20 representing each rearing system (extensive, semi-intensive, and intensive).

The researchers were trained in data collection, questionnaire administration, description of each question, data collection ethics, and consent. A questionnaire was administered to the farmers through face-to-face interviews. The data collection tool for respondents was prepared using the Google Form Platform.

### Questionnaire design and measurement

A structured questionnaire was developed and reviewed by experts to ensure its consistency and validity. Three experts in this field evaluated the index of item-objective congruence (IOC), and the overall IOC value of the KAP questionnaire was 0.8.

Data collection was conducted through face-to-face interviews in Thai, after which the questionnaire was translated into English and included in the Supplementary Material. The questionnaire comprised two main sections: (1) sociodemographic characteristics, including age, gender, educational level, source of cattle income, farming experience, annual household income, and herd size and (2) KAP assessment of animal welfare principles.

(1) *Knowledge assessment:* The knowledge section consisted of 25 questions covering key aspects of animal welfare principles, including good feeding, good housing, good health, good behavior, and stockpersonship. Each correct response was awarded 1 point, and incorrect answers received 0 points, resulting in a maximum possible score of 25. Levels of knowledge were categorized into 3 groups using an equal interval classification: poor knowledge (0–8 points), moderate knowledge (9–16 points), and good knowledge (17–25 points).

(2) *Attitude assessment:* The attitude section comprised 25 questions designed to assess farmers’ perceptions and opinions regarding animal welfare across 5 key domains: good feeding, good housing, good health, good behavior, and stockpersonship. Responses were measured using a 5-point Likert scale, coded as follows: strongly agree (5), agree (4), neutral (3), disagree (2), and strongly disagree (1).

The minimum possible score was 25 (if all responses were “strongly disagree”), and the maximum possible score was 125 (if all responses were “strongly agree” = 5 × 25). The equal interval method was used to classify attitude levels into 3 categories: negative (25–58 points), neutral (59–91 points), and positive (92–125 points).

(3) *Practice assessment:* The 25 questions in the practice section evaluate daily farm activities and adherence to exceptional concepts of animal welfare within the 5 fundamental areas: good feeding, good housing, good health, good behavior, and stockpersonship. The codes and categorization of the responses were as follows: usually practiced (2), sometimes practiced (1), and never practiced (0).

The minimum possible score was 0 (if all responses were “never practice”), and the maximum possible score was 50 (if all responses were “usually practice” = 2 × 25). The equal interval method was applied to classify practice levels into 3 categories: poor practice (0–16 points), good practice (17–33 points), and very good practice (34–50 points).

This structured approach ensures an objective and systematic measurement of farmers’ KAPs related to animal welfare in beef cattle farming.

### Data collection

Data were collected through structured face-to-face interviews with beef cattle farmers in Phayao Province, Thailand, between July and November 2024. The interviews were conducted in the Thai language to ensure clarity and ease of communication for the respondents.

A representative sample that represented three farming practices was obtained through the selection of farmers using the purposive sampling method, whereby farmers within all three farming systems were sampled. The study was limited by the inclusion criteria to comprise farmers who actively participated in any of the three farming systems (intensive, semi-intensive, and extensive) of farming, had at least one year of experience, and were willing to participate. Interviews were conducted by skilled interviewers who were experts in cattle research and survey methods. The sociodemographic background, as well as the farmers’ KAPs toward the concepts of animal welfare, was discussed during a roughly 30 to 45-min interview.

To reduce bias, interviewers provide the same clarification to all farmers. The interviewers were trained in ways to avoid biases that could influence the way the respondents answered their questions. Besides, anonymity and voluntary disclosure encouraged open and non-biased responses. We documented the received information in both digital and manual forms, comparing it to ensure its completeness. Furthermore, the methodology has provided a convenient and accommodating solution, which can reasonably be customized to the real-life scenarios of farming where the data are collected.

### Data analysis

Data were imported into a Microsoft Excel spreadsheet to be cleaned and coded and then transferred to SPSS and R statistical analysis software. The demographic data and KAP levels among beef cattle farmers were summarized using descriptive statistics. Pearson’s chi-square tests were conducted to identify significant determinants influencing KAPs related to animal welfare principles. A 5% significance level was set, and all tests were conducted as two-tailed. The data were normally distributed, as determined by Shapiro–Wilk’s test of normality (*p *> 0.05). Additionally, a multivariate analysis of variance (MANOVA) was performed to assess the relationships between the dependent variables (KAPs) and the independent variable (farming system). By analyzing a linear combination of these numerical variables, this method was used to determine whether significant differences existed among the three farming systems regarding farmers’ KAPs. Furthermore, Pearson’s correlation test was conducted to examine the relationships between KAP scores.

## Results and Discussion

Table 1 presents Cronbach’s alpha coefficients for the KAP components related to animal welfare principles. The knowledge scale, which included 25 items, demonstrated good internal consistency, with a Cronbach’s alpha of 0.81, indicating acceptable reliability. Similarly, the attitude scale, composed of 25 items, exhibited high reliability (α = 0.94), demonstrating strong internal consistency among its components. The practices scale, which contained 25 items, showed excellent reliability (α = 0.96). The overall reliability coefficient for all 75 items was 0.90, confirming the high level of internal consistency in the questionnaire.

These findings indicate that the questionnaire implemented in this study was highly reliable for assessing farmers’ KAPs related to animal welfare principles. A Cronbach’s alpha above 0.70 is generally regarded as acceptable, while values exceeding 0.80 and 0.90 indicate good and excellent reliability, respectively [11]. This study satisfied these criteria by proving the reliability and internal consistency of the measuring scales. Chan and Idris [12] found that a greater Cronbach’s alpha coefficient increases confidence in the reliability of research tools, therefore ensuring that the obtained data reflects the intended construction.

Table 2 presents the sociodemographic characteristics of beef cattle farmers categorized into extensive, semi-intensive, and intensive farming systems. The majority of participants were male (63.33%), with the highest proportion observed in the intensive system (75.00%). Age distribution revealed that most farmers were over 60 years old (43.33%), followed by 51–60 years (35.00%), and only 6.67% were between 21 and 40 years old. Education levels varied, with 58.33% having a primary education, 26.67% completing high school, and 11.67% achieving higher education, primarily within the intensive system (35.00%). These results are consistent with Özdemir et al. [13], who found that 33.5% of livestock farmers had completed secondary education and 34.4% had only a primary education. Limited formal education could limit access to modern farming technologies and best practices, consequently influencing production and innovation. These results emphasize the importance of more effectively adopting welfare-friendly and sustainable farming techniques through the implementation of increased access to agricultural education and training programs. Cattle farming supplied supplementary income to 73.33% of farmers; however, in the intensive system, 45.00% depended on cattle as their primary source of income. Of the farmers, 70% had more than 6 years of experience, with the highest levels observed across all 3 systems. Household income levels varied, with 50.00% of farmers earning between THB 10,000 and 50,000 per year, while 18.33% earned over THB 150,000, predominantly within the intensive system (45.00%). Herd size distribution varied significantly among farming systems. More than 10 cattle were managed by 61.67% of farmers, particularly within the extensive system (80.00%), whereas smaller herds of 6–10 cattle were more common in the intensive system (30.00%). These findings indicate that intensive farming systems are associated with more educated farmers, higher income levels, and greater dependence on cattle as a primary income source. In contrast, extensive and semi-intensive systems were characterized by older farmers with lower formal education and smaller-scale operations.

**Table 1.** Cronbach’s alpha for animal welfare knowledge, attitude, and practice items.

Variables	Number of questions	Cronbach’s α
Knowledge questions	25	0.81
Attitude questions	25	0.94
Practice questions	25	0.96
Overall	75	0.90

**Table 2.** Sociodemographic characteristics of participants in the three rearing systems (*n* = 60).

Variables	Categories	Extensive (*n* = 20)		Semi-intensive (*n* = 20)		Intensive (*n* = 20)		Overall (*n* = 60)	
		N	(%)	N	(%)	N	(%)	N	(%)
Gender	Female	9	45.00	8	40.00	5	25.00	22	36.67
	Male	11	55.00	12	60.00	15	75.00	38	63.33
Age (Year)	21–40	0	0.00	1	5.00	3	15.00	4	6.67
	41–50	3	15.00	1	5.00	5	25.00	9	15.00
	51–60	7	35.00	10	50.00	4	20.00	21	35.00
	>60	10	50.00	8	40.00	8	40.00	26	43.33
Education level	No formal education	1	5.00	1	5.00	0	0.00	2	3.33
	Primary school	14	70.00	13	65.00	8	40.00	35	58.33
	High school	5	25.00	6	30.00	5	25.00	16	26.67
	Above	0	0.00	0	0.00	7	35.00	7	11.67
Source of raising cattle income	Main income	2	10.00	5	25.00	9	45.00	16	26.67
	Supplement income	18	90.00	15	75.00	11	55.00	44	73.33
Farm experience (Years)	1–3	1	5.00	0	0.00	2	10.00	3	5.00
	4–6	4	20.00	6	30.00	5	25.00	15	25.00
	>6	15	75.00	14	70.00	13	65.00	42	70.00
Household income (Baht/year)	10,000–50,000	11	55.00	13	65.00	6	30.00	30	50.00
	50,000–100,000	5	25.00	4	20.00	3	15.00	12	20.00
	100,000–150,000	3	15.00	2	10.00	2	10.00	7	11.67
	>150,000	1	5.00	1	5.00	9	45.00	11	18.33
Herd size (Heads)	1–5	2	10.00	3	15.00	1	5.00	6	10.00
	6–10	2	10.00	9	45.00	6	30.00	17	28.33
	>10	16	80.00	8	40.00	13	65.00	37	61.67

The *chi*-square test revealed significant differences (*p* < 0.001) in all variables; therefore, the association between farming systems (extensive, semi-intensive, and intensive) and KAPs related to animal welfare was assessed among the 60 participants (Table 3). Farmers in the intensive system demonstrated the highest levels of knowledge, with 100.00% categorized as having good knowledge, compared to 95.00% in the semi-intensive system and only 55.00% in the extensive system, with only 45.00% having moderate knowledge. This trend suggests that farmers in intensive systems have greater exposure to best practices, training, and regulatory compliance measures.

Attitude levels varied among farming systems, with 55.00% of intensive system farmers displaying a positive attitude toward animal welfare, while 95.00% of semi-intensive farmers exhibited a neutral attitude. In contrast, 90.00% of farmers in the extensive system demonstrated negative attitudes, highlighting potential knowledge gaps and resistance to welfare adoption. These findings contrast with those of Spooner et al. [14], who found that Canadian beef farmers in extensive systems were aware of the importance of animal welfare and linked it to health, comfort, and natural behaviors. However, the views of farmers on animal welfare depend on personal experience and local pressure that contribute to differing perspectives on welfare practices. Some of the differences noted in this research could be explained by the differences in policy implementation and incentives in the marketplace, as well as the sociocultural understanding of animal treatment in different areas.

The maximum percentage of farmers who demonstrated very good practices was in intensive systems (80.00%), whereas 95.00% of farmers in semi-intensive systems demonstrated good practices [15]. Conversely, 95.00% of the farmers in extensive systems were rated as having poor practices, meaning that they lacked adherence to animal welfare standards, as is common in traditional farms. This pattern was also related to educational background, as 11.67% of the respondents, and especially those who were in the intensive system (35.00%), had higher education qualifications. The findings are compared with those of Demera et al. [16], which indicate that farmers with higher education degrees are more compliant with hygienic practices, animal welfare standards, and environmental control strategies, showing the role of education in enhancing the quality of farms and sustainability. Consistent with Islam and Ahmed [17], the results of the KAPs of different stakeholders regarding zoonotic diseases in the identified coastal regions of the Barguna District, Bangladesh, indicated that individuals with low levels of formal education or no formal education had significantly less knowledge about zoonotic diseases. This evidence attests to the value of education in increasing knowledge about health-related risks as far as animals are concerned. Debnath and Paul [18] evaluated farmers’ knowledge of animal anesthetic use and animal anesthetics. Their study signified the necessity of training programs aimed at developing the knowledge of farmers regarding the role of anesthesia because potential knowledge growth may serve as the source of better decisions concerning the health and welfare of animals, particularly during procedures. These results further confirm the effectiveness of education in improving animal welfare practices overall and enhancing outcomes in livestock management specifically.

**Table 3.** Chi-square test results for farming systems based on the level of KAPs regarding animal welfare principles (*n* = 60).

Variables	Extensive (*n* = 20)	Semi-intensive (*n* = 20)	Intensive (*n* = 20)	*p*-value
Knowledge level				
Good	11 (55.00%)	19 (95.00%)	20 (100.00%)	< 0.001
Moderate	9(45%)	1(5%)	–	
Attitudes level				
Positive	–	–	11 (55.00%)	< 0.001
Neutral	2 (10.00%)	19 (95.00%)	9 (45.00%)	
Negative	18 (90.00%)	1 (5.00%)	–	
Practices level				
Very good	–	1 (5.00%)	16 (80.00%)	< 0.001
Good	1 (5.00%)	19 (95.00%)	4 (20.00%)	
Poor	19 (95.00%)	–	–	

These results explain that the nature of the farming system plays an important role in determining KAPs of farmers in animal welfare. They were more conscious and implemented welfare standards in intensive types of farming compared to less intensive farmers, perhaps due to higher education levels, production market incentives, and access to training programs. Meanwhile, extensive systems were less compliant with the principles of animal welfare, which would require special interventions, training of farmers, and policies to close the welfare gap and facilitate useful practices in all systems.

Table 4 presents the descriptive statistics of KAP scores according to farming systems and reveals considerable differences in the perceptions and implementation of animal welfare concepts among farmers. All parts of KAP (knowledge, 23.25 ± 1.80; attitudes, 91.85 ± 1.45; and practices, 39.65 ± 5.57) were higher in the intensive farming system, followed by the semi-intensive system, which had lower scores in all areas compared to the intensive system, and the extensive system had the lowest scores overall in the study. The extensive system recorded the lowest average scores, which were mainly in practice (11.25 ± 1.67), and possibly, there may be a problem with the adoption of welfare-friendly practices occasioned by limited knowledge, resources, and infrastructure. The KAP scores indicate that the transition to the intensive system enhances management and education levels and resource access, which are critical to enhancing knowledge and practice that comes with animal welfare. This conclusion agrees with the findings of Zoltick et al. [19], who reviewed how pain affects cattle and the potential for pain relief treatments, highlighting the importance of targeted pain management and recognizing pain signs to improve animal welfare.

**Table 4.** Descriptive statistics of knowledge, attitude, and practice scores across farming systems (*n* = 60).

Farming systems		Mean	S.D.	*N*
Knowledge score	ExtensiveSemi-intensive IntensiveTotal	16.30 21.15 23.25 20.23	3.41 2.85 1.80 4.00	20 20 20 60
Attitude score	Extensive Semi-intensive Intensive Total	54.15 74.80 91.85 73.60	5.57 5.60 4.51 16.38	20 20 20 60
Practice score	Extensive Semi-intensive Intensive Total	11.25 21.70 39.65 24.20	2.67 4.62 5.57 12.61	20 20 20 60

Nonetheless, one of the greatest barriers to the improvement of farm welfare standards is poor infrastructure, including insufficient facilities for pain management and unsuitable handling systems. Likewise, Alquati et al.’s [20] survey of biosecurity and animal welfare of beef cattle farms revealed that many farmers are not adequately trained in the aspects of animal welfare, thus hindering their prospects of taking appropriate animal welfare measures on their farms.

These results support the need to use special training programs and policy measures to promote animal welfare education and experience, especially in extensive and semi-intensive systems among farmers. Overcoming infrastructural constraints, skill reinforcement, and enhanced welfare-compliant approaches to the business are important to ensure sustainable and ethical livestock production.

We performed a MANOVA to determine statistically significant changes in the overall KAP scores regarding animal welfare principles among beef cattle farmers practicing diverse farming systems (extensive, semi-intensive, and intensive). MANOVA was chosen because it provides a concurrent examination of numerous dependent variables while considering their potential associations, thereby offering in-depth insight into the impact of farming methods on KAP components. The investigation aimed to determine whether the farming system type significantly influenced farmers’ KAPs of animal welfare principles. Before performing the MANOVA, we assessed the assumptions of equality of variance–covariance matrices using Box’s M test and the equality of error variances with Levene’s test. Despite the significant finding in Box’s M test (*p* = 0.003), indicating a violation of homogeneity, the analysis was considered valid due to the approximately equal sample sizes. Levene’s test revealed significant differences in the variances for knowledge (*F* = 5.579, *p *= 0.006) and practices (*F* = 5.302, *p *= 0.008), indicating that these variables had heterogeneous variances across farming systems. In contrast, attitudes (*F* = 0.017, *p* = 0.984) met the assumption of homogeneity of variance, suggesting that responses to attitudes were more consistent across groups (Table 5).

These findings suggest that the implementation of animal welfare knowledge and practices varies significantly among farming systems, likely because of differences in education, experience, resource availability, and farm management styles. The significant variance in knowledge and practices indicates unequal exposure to information and varying adoption of welfare-friendly management approaches, which aligns with the expectation that intensive farming systems exhibit better compliance than extensive farming systems. Although the variance assumptions were not fully met, MANOVA can still provide meaningful insights into how different farming systems influence KAP regarding animal welfare with adjustments in interpretation.

**Table 5.** Tests for equality of variances and covariances in MANOVA analysis.

Multivariate test of Homoscedasticity				
**Box's Test of Equality of Covariance Matrices**	**Box's M**	***F***	***p*-value**	
	31.875	2.452	0.003	
Univariate test of Homoscedasticity				
Levene’s test of equality of error variances				
**Dependent variables**	***F***	**df1**	**df2**	***p*-value**
Knowledge	5.579	2	57	0.006
Attitudes	0.017	2	57	0.984
Practices	5.302	2	57	0.008

**Table 6.** Statistical comparison of knowledge, attitude, and practice scores using MANOVA.

Multivariate test				
Statistic test	Value	*F*	*p*-value	
Wilks' Lambda	0.059	57.445	0.000	
Univariate test				
**Dependent variables**	**SS**	**MS**	**F**	***p*-value**
Knowledge	508.233	254.117	33.032	0.000
Attitudes	14256.100	7128.050	258.082	0.000
Practices	8253.100	4126.550	207.694	0.000

Table 6 presents the results of the MANOVA, examining the differences in KAP scores across farming systems. The Wilks’ Lambda statistic (0.059, *F* = 57.445, *p* < 0.001) indicates a significant multivariate effect, confirming that farming system type had a substantial impact on the combined KAP scores.

Univariate analysis of variance (ANOVA) further revealed statistically significant differences across farming systems for each dependent variable. The knowledge score (*F* = 33.032, *p *< 0.001) showed significant variation, suggesting that knowledge levels differed considerably according to the type of farming system used. Similarly, the attitude (*F* = 258.082, *p* < 0.001) and practice scores (*F* = 207.694, *p* < 0.001) demonstrated highly significant differences, indicating that farmers’ perceptions and implementation of animal welfare practices varied substantially, depending on their farming system.

These results confirm the assumption that farmers, via different management systems, have different KAP levels related to animal welfare. The higher *F*-values for attitudes and practices indicate that the farming system type, possibly caused by structural differences, access to resources, and training opportunities, influences these features. Overall, the findings highlight the need for focused interventions and education programs to improve animal welfare compliance, especially in extensive and semi-intensive farming systems, in which gaps in knowledge and practice implementation are apparent.

We used Pearson’s correlation analysis to determine the strength and direction of the linear correlations between beef cattle farmers’ knowledge, attitudes, and behaviors related to animal welfare. This investigation aimed to determine whether higher knowledge levels correlated with positive attitudes and whether both knowledge and attitudes were related to improved welfare practices on farms. Identifying these relationships can provide valuable data on the overall impacts of these factors as well as potential areas where interventions can be implemented to maximize animal welfare. These significant positive relationships indicate that it is possible to improve welfare practices directly by enhancing the knowledge and attitudes of farmers.

**Figure 2.**
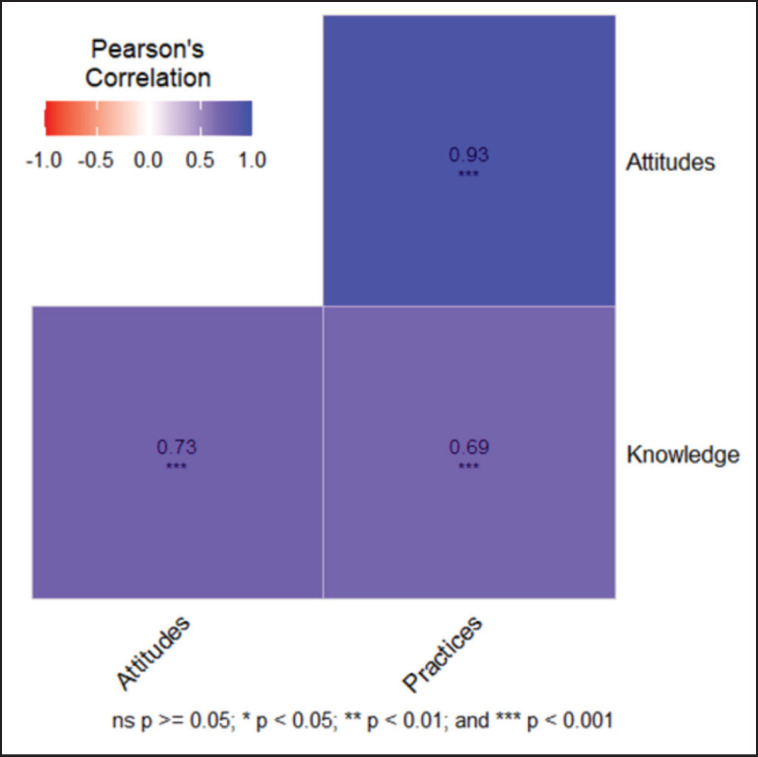
Correlation analysis of KAPs of all respondents.

Pearson correlation coefficients measure how attitudes and practices (A-P), knowledge and attitudes (K-A), and knowledge and practices (K-P) are related to each other in the entire study group, as shown in Figure 2. The results showed a highly significant positive value in A-P (0.93, *p* < 0.001), which means that farmers with a greater positive attitude toward animal welfare have better tendencies to use better animal welfare standards. The findings correspond with those of Salvin et al. [21], who surveyed Australian cattle farmers to explore the acceptability and feasibility of animal welfare practices within farmer-led beef production systems. They found that the attitudes and beliefs of farmers play a key role in their readiness to accept and implement the facilitation of better welfare; the more positive the attitude toward animal welfare, the more willing the farmer is to cooperate and implement new methods of management on the farms.

Additionally, a high positive correlation was observed in K-A (0.73, *p* < 0.001), which implies that the higher the level of knowledge, the more positive attitudes became. The finding suggests that training courses and education programs can shape positive perceptions of welfare-friendly policies. Furthermore, there was a moderately crucial positive correlation in K-P (0.69, *p* < 0.001), which proved that improved insight leads to enhanced enforcement of welfare practices on farms. Such findings indicate a high KAP in animal shelter adoption. Enhancing the practical realization of welfare-oriented policies is based on raising the knowledge and attitudes of farmers. Therefore, sustainable and ethical production of beef cattle encompasses the use of targeted education programs, policy promotion, and changes in attitudes and practices.

## Conclusion

This study examined the differences in KAPs regarding animal welfare principles among beef cattle producers in Thailand’s Phayao Province based on the extensive, semi-intensive, and intensive farming practices. The results showed that farmers using intensive farming systems were better at welfare practices, more knowledgeable, and had more positive attitudes than those using extensive and semi-intensive systems, which had welfare practice issues. The KAP’s components were interconnected, and encouraging farmers to have positive attitudes and knowledge may be the best way to increase welfare practices. Additionally, in less intense farming systems, challenges include inadequate facilities, limited knowledge, and fundamental traditional practices that need to be addressed as awareness of animal welfare increases. The MANOVA results confirmed the significant influence of the type of farming system on KAP levels, highlighting the importance of focused actions to address these differences. This study emphasizes the necessity of policy-driven actions, farmer education programs, and investment in farm infrastructure to promote sustainable beef cattle production and encourage the acceptance of welfare-friendly methods. Future strategies require the integration of scientific information with actual on-farm programs, the promotion of optimal procedures through focused training, and market-driven promotions that align economic sustainability with animal welfare. By addressing these crucial problems, the cattle farming business will continue toward a more sustainable and ecologically friendly production method that satisfies global welfare requirements and improves customer expectations.

## List of Abbreviations

MANOVA, Multivariate analysis of variance; KAP, knowledge, attitudes, and practices; R, statistical programming language; SPSS, statistical package for the social sciences; THB, Thai Baht.
